# Childhood cancer and future cardiometabolic health

**DOI:** 10.1038/s44324-026-00128-y

**Published:** 2026-09-29

**Authors:** Abel Plaza-Florido, Pedro Carrera-Bastos, Shlomit Radom-Aizik, Inmaculada Pérez-Prieto, Francesca Lanfranconi, Gonzalo Saco-Ledo, Raquel Revuelta-Iniesta, Kirsten K. Ness, Borja del Pozo Cruz, Carmen Fiuza-Luces, Alejandro Lucia

**Affiliations:** 1 Research Center for Exercise Medicine and Sleep/Pediatric Exercise and Genomics Research Center, Department of Pediatrics, School of Medicine, University of California Irvine, Irvine, CA USA; 2 Faculty of Biomedical and Health Sciences, Universidad Europea de Madrid, Villaviciosa de Odón, Madrid, Spain; 3 Nutrition Laboratory, Faculty of Medicine, Academic Center of Medicine of Lisbon, University of Lisbon, Lisbon, Portugal; 4 Center for Primary Health Care Research, Department of Clinical Sciences in Malmö, Lund University, Malmö, Sweden; 5 IVIRMA Global Research Alliance, IVI Foundation, Instituto de Investigación Sanitaria La Fe (IIS La Fe), Valencia, Spain; 6 Centro Maria Letizia Verga, Fondazione Monza e Brianza per il Bambino e la sua Mamma, Monza, Italy; 7 Department of Sport Sciences, Faculty of Medicine, Health Sciences, and Sports, Universidad Europea de Madrid, Villaviciosa de Odón, Madrid, Spain; 8 Department of Public Health and Sports Sciences, Medical School, Children’s Health and Exercise Research Centre, University of Exeter, Exeter, UK; 9 Child Life and Health, University of Edinburgh, Edinburgh, UK; 10 Department of Epidemiology and Cancer Control, St. Jude Children’s Research Hospital, Memphis, TN USA; 11 Research Institute of the Hospital 12 de Octubre (‘imas12’), Madrid, Spain

**Keywords:** Cancer, Diseases, Oncology

## Abstract

Childhood cancer survivors face an elevated risk of cardiometabolic disease that can emerge years after treatment completion. This Review summarises the mechanisms underlying this vulnerability, the contribution of biological, social, and lifestyle factors across the life course, and emerging opportunities for early prevention and interventions to improve long-term cardiometabolic health.

## Introduction

Medical advances have led to substantial improvements in survival from childhood cancer. Today, more than 80% of children diagnosed with cancer in high-income countries can expect to survive ≥ 5 years beyond their diagnosis^1,2^. This expanding population of survivors represents a major public health achievement but also brings increased attention to the consequences of intensive treatments, particularly long-term cardiometabolic complications^3,4^.

The aim of this review is to provide an updated synthesis of cardiometabolic health in childhood cancer survivors. After summarising the cardiovascular and metabolic consequences of paediatric cancer therapy, we describe the cardiometabolic profile of childhood cancer survivors, as well as the ethnic, social, genetic, and sex-related determinants that shape this profile. We further address lifestyle patterns that are likely to influence cardiometabolic risk and discuss the potential benefits of lifestyle-based interventions in improving long-term health outcomes. Because cancer treatment occurs during critical windows of cardiovascular and metabolic development, treatment-related insults may reshape cardiometabolic trajectories decades before overt disease becomes clinically evident.

## Treatment-related exposures and mechanistic cardiometabolic injury

### Anthracyclines and chemotherapy‑related cardiometabolic injury

**Fig. 1:**
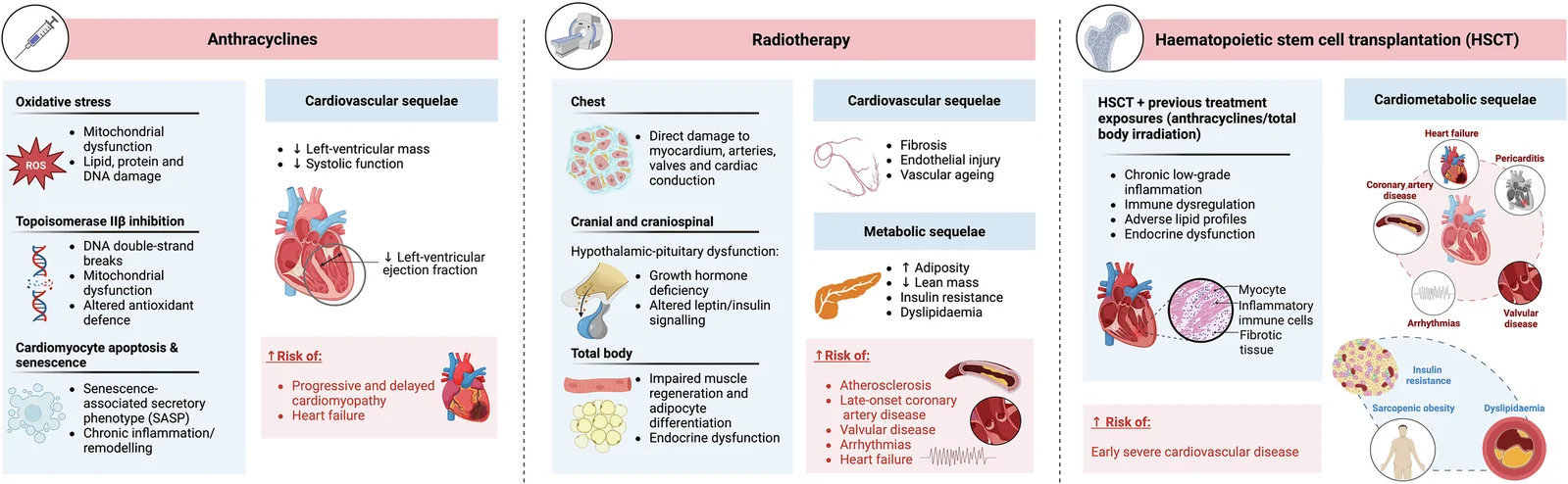
Mechanisms and long-term cardiometabolic sequelae of cancer therapies in childhood and adolescence. Cancer therapies commonly used during childhood and adolescence can cause long-term cardiovascular and metabolic complications through distinct but overlapping mechanisms. Anthracyclines induce oxidative stress, topoisomerase IIβ inhibition, and cardiomyocyte apoptosis and senescence, leading to structural and functional cardiac abnormalities and an increased risk of cardiomyopathy and heart failure. Radiotherapy can damage the heart and vasculature, disrupt hypothalamic–pituitary function, and alter whole-body metabolism, increasing the risk of atherosclerosis, coronary artery disease, valvular disease, arrhythmias, and heart failure. Haematopoietic stem cell transplantation (HSCT), particularly when combined with prior chemotherapy or total body irradiation, contributes to chronic inflammation, immune dysregulation, endocrine dysfunction, and adverse metabolic profiles, resulting in elevated risks of early-onset cardiovascular disease and metabolic disorders. This figure was created with BioRender. ROS reactive oxygen species, HSCT haematopoietic stem cell transplantation.

Anthracyclines remain a cornerstone of paediatric oncology but are among the most cardiotoxic agents used in childhood cancer treatment. In addition to the well‑established, dose‑dependent impairments in left‑ventricular mass and systolic function observed during and shortly after therapy, anthracyclines initiate a cascade of molecular events that predispose to progressive and often delayed cardiomyopathy (Fig. 1). Central to this process is the generation of reactive oxygen species (ROS) through redox cycling of the anthracycline quinone moiety, leading to oxidative damage of lipids, proteins, and mitochondrial DNA in cardiomyocytes^3,5,6^.

A second, now well‑established mechanism involves inhibition of topoisomerase IIβ, a key enzyme required for maintaining genomic stability in terminally differentiated cardiomyocytes. Anthracycline‑mediated topoisomerase IIβ poisoning results in DNA double‑strand breaks, altered transcription of mitochondrial biogenesis and antioxidant genes, and activation of apoptotic pathways^7,8^. These processes converge on mitochondrial dysfunction, characterised by impaired oxidative phosphorylation, reduced ATP generation, increased mitochondrial fragmentation, and long‑lasting deficits in myocardial energy homoeostasis—features consistently observed in experimental and translational models of anthracycline cardiotoxicity^3,9,10^.

Importantly, anthracycline‑induced injury may remain clinically silent for many years. Subclinical myocardial damage accumulated during childhood can later decompensate in the presence of age‑related haemodynamic stressors or acquired cardiometabolic risk factors. Indeed, obesity, insulin resistance, hypertension, and dyslipidaemia potentiate oxidative stress, systemic inflammation, and increased myocardial workload, biologically amplifying anthracycline‑related cardiac injury^9^. This interaction provides a mechanistic basis for the markedly elevated risk of heart failure observed in long‑term survivors exposed to high cumulative anthracycline doses who subsequently develop adverse cardiometabolic profiles^11,12^.

### Radiotherapy and disruption of developmental cardiometabolic pathways

Radiotherapy administered during childhood exerts diverse effects on cardiovascular and metabolic development, particularly when involving the chest or central nervous system. Chest‑directed radiotherapy causes direct damage to myocardial tissue, coronary arteries, cardiac valves, and the cardiac conduction system, promoting fibrosis, microvascular rarefaction, endothelial dysfunction, and accelerated atherosclerosis^3,13,14^. These structural and functional alterations often progress insidiously, laying the groundwork for late‑onset coronary artery disease, valvular dysfunction, arrhythmias, and heart failure decades after exposure.

Beyond its direct cardiac effects, radiotherapy can disrupt critical neuroendocrine pathways that regulate growth and metabolic homoeostasis. Cranial and craniospinal irradiation frequently impair hypothalamic–pituitary function, leading to growth hormone deficiency, altered leptin and insulin signalling, and dysregulation of appetite and energy balance^15–17^. Such endocrine sequelae are strongly associated with increased adiposity, reduced lean mass, insulin resistance, and dyslipidaemia, even among survivors whose body mass index (BMI) remains within the normal range^18^. Notably, total body irradiation—commonly used as part of conditioning regimens before haematopoietic stem cell transplantation (HSCT)—emerges as one of the strongest predictors of long‑term metabolic dysfunction^18^.

Radiotherapy‑induced endothelial injury may further link cardiovascular and metabolic sequelae. Survivors exposed to radiotherapy exhibit increased carotid and femoral intima‑media thickness, alongside persistent activation of haemostatic and inflammatory pathways, indicative of premature vascular ageing^14^. These vascular changes may interact synergistically with metabolic abnormalities to accelerate cardiometabolic disease across the life course.

### Haematopoietic stem cell transplantation and systemic metabolic injury

HSCT represents a life‑saving therapy for many children with high‑risk malignancies but is associated with a particularly high burden of late cardiometabolic morbidity. HSCT survivors are exposed to multiple, often synergistic insults, including high‑dose chemotherapy, total body irradiation, prolonged corticosteroid therapy, and immune‑mediated complications such as graft‑versus‑host disease^19^. Together, these factors promote widespread metabolic and cardiovascular dysfunction.

Long‑term follow‑up studies consistently show that HSCT survivors display impaired insulin sensitivity, adverse lipid profiles, and profound alterations in body composition, characterised by increased visceral adiposity and reduced lean mass despite relatively preserved BMI^18^. This sarcopenic‑obese phenotype is metabolically deleterious, strongly linking HSCT to insulin resistance, chronic low‑grade inflammation, and reduced physical capacity. Total body irradiation appears to be a central mechanistic driver of these changes, plausibly through combined effects on skeletal muscle regeneration, adipocyte differentiation, and endocrine signalling^18^, thereby promoting insulin resistance and long-term cardiometabolic dysfunction^20^.

In parallel, HSCT survivors exhibit a markedly elevated prevalence of severe cardiovascular disease at young ages, reflecting both direct myocardial injury and the cumulative impact of systemic metabolic derangements^19^. Chronic inflammation, immune dysregulation, and premature frailty may further compound cardiometabolic risk, placing HSCT survivors among the highest‑risk subgroups within childhood cancer survivorship^19^.

## Long‑term cardiometabolic phenotype and disease trajectory in survivors

### Accelerated cardiometabolic ageing and late mortality

**Fig. 2:**
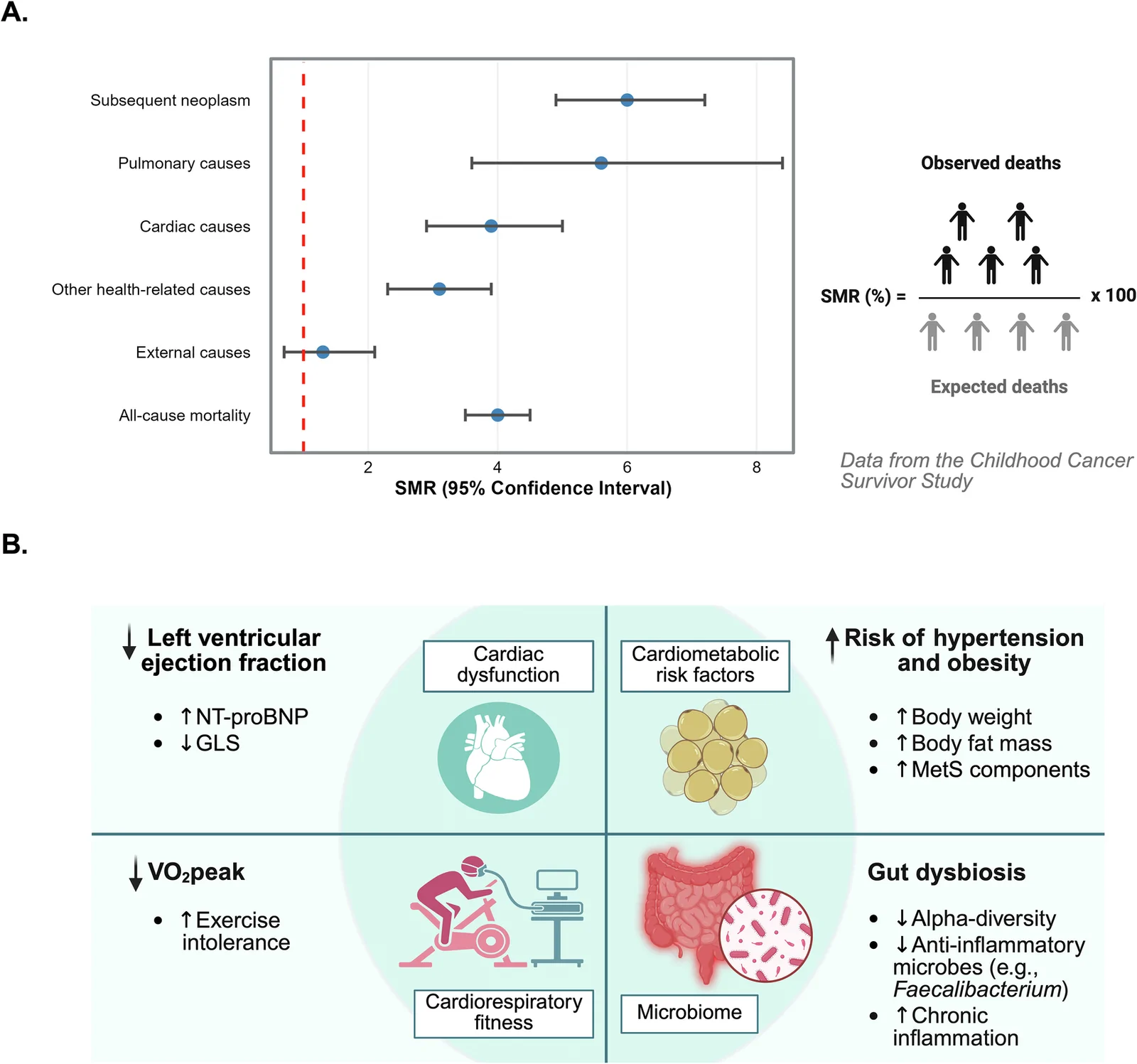
Late mortality and integrated cardiometabolic profile in survivors of childhood cancer. **A** Causes of late mortality at 40 years after childhood cancer diagnosis in survivors from North America compared with the general population. **B** Integrated cardiometabolic profile of childhood cancer survivors, spanning cardiac dysfunction (reduced left‑ventricular ejection fraction, together with raised NT‑proBNP, a marker of myocardial wall stress, and reduced GLS, an early indicator of impaired systolic deformation), cardiometabolic risk factors (increased body weight and fat mass and metabolic syndrome components), reduced cardiorespiratory fitness with exercise intolerance, and gut dysbiosis with lower alpha‑diversity and depletion of anti‑inflammatory, butyrate‑producing taxa such as *Faecalibacterium*. This figure was created with BioRender. GLS global longitudinal strain, MetS metabolic syndrome, NT-proBNP N-terminal pro-B-type natriuretic peptide, SMR standardised mortality ratio, VO2peak peak oxygen uptake (typically assessed during cardiopulmonary exercise testing until volitional exhaustion).

Long‑term survivors of childhood cancer experience a substantially increased burden of chronic cardiometabolic disease and premature mortality compared with the general population. In a large North American cohort of 34,230 5‑year survivors, cumulative all‑cause mortality reached 23.3% by 40 years after diagnosis, far exceeding age‑matched expectations ( < 5%) in the general population (Fig. 2A)^21^. As survivors age, cardiovascular and metabolic conditions contribute an increasing proportion of excess late mortality, reflecting a shift from cancer‑related to treatment‑ and lifestyle‑associated causes of death^21^.

Compelling evidence supports the concept of accelerated cardiometabolic ageing in this population. A recent analysis combining data from the Childhood Cancer Survivor Study and national databases showed that childhood cancer survivors develop major chronic conditions—including coronary artery disease, heart failure, myocardial infarction, stroke, and valvular disease—approximately 18 years earlier than individuals without a cancer history^22^. By the fifth decade of life, 20% of survivors had already experienced at least one major cardiometabolic condition, whereas a comparable disease burden in the general population typically emerged closer to age 65^22^. Prospective data from the St. Jude Lifetime Cohort Study corroborate these findings, revealing a cumulative incidence of major adverse cardiovascular events of 17.7% among survivors, compared with only 0.9% in community controls (median follow‑up, 20.3 years)^23^.

Despite this elevated baseline risk, cardiometabolic trajectories among survivors remain, at least in part, modifiable. A healthy lifestyle—defined by regular physical activity, absence of smoking, and freedom from hypertension and diabetes—was independently associated with a 20–30% reduction in health‑related mortality, irrespective of cancer type or treatment exposure^21^. These observations indicate that treatment‑related biological injury initiates vulnerability, but downstream cardiometabolic outcomes are shaped by cumulative exposures and behaviours across the life course.

### Cardiac dysfunction across the life course

Cardiac dysfunction is a central component of the long‑term cardiometabolic phenotype observed in childhood cancer survivors. Meta‑analytical evidence shows that survivors have persistently lower left‑ventricular systolic function than age‑matched individuals without a cancer history, with a pooled mean difference of −1.61% in left‑ventricular ejection fraction (Fig. 2B)^24^. Although modest at the population level, such reductions likely reflect diminished cardiac reserve, predisposing survivors to earlier clinical decompensation as haemodynamic demands increase with ageing or superimposed cardiometabolic stressors.

Beyond systolic dysfunction, autonomic cardiovascular abnormalities are common. In the St. Jude Lifetime Cohort Study, 24.3% of survivors met criteria for cardiac autonomic dysfunction—defined by abnormalities in resting heart rate, heart rate reserve, blood pressure response to exercise, or heart rate recovery—compared with 6.6% of controls^25^. Exposure to chest‑directed or cranial radiotherapy and to carboplatin was associated with greater autonomic impairment, suggesting combined contributions of direct cardiac injury and central nervous system effects^25^. Such dysregulation may contribute to exercise intolerance, arrhythmogenic risk, and adverse cardiovascular events later in life.

Emerging evidence supports the value of early cardiac biomarkers in detecting subclinical myocardial injury well before overt cardiomyopathy develops. In anthracycline‑exposed survivors from the St. Jude Lifetime Cohort Study, elevated N‑terminal pro-B-type natriuretic peptide (NT‑proBNP) and abnormal global longitudinal strain (GLS) independently predicted incident cardiomyopathy over a median follow‑up of 5.2 years^26^. Survivors with abnormal values of both markers had a fourfold increased risk, which rose to 14-fold among those exposed to high cumulative anthracycline doses ( ≥ 250 mg/m²) without radiotherapy, even in the presence of preserved ejection fraction^26^. Complementing these findings, serum proteomics analyses identified a multi‑protein signature, derived in survivors with subclinical cardiomyopathy, that discriminated severe cardiomyopathy in an independent sample with over 80% classification accuracy^27^.

### Cardiorespiratory fitness as an integrative cardiometabolic phenotype

Cardiorespiratory fitness (CRF) integrates the functional capacity of the cardiovascular, pulmonary, muscular, and metabolic systems and is recognised by the American Heart Association as a key clinical vital sign^28^. In childhood cancer survivors, impairments in CRF appear early and frequently persist across survivorship. Within the first month of treatment, children and adolescents with newly diagnosed cancer already exhibit markedly reduced CRF compared with healthy peers, with a mean difference approaching −20 mL/kg/min^29^.

Long‑term deficits in CRF are well documented. A meta‑analysis including 786 survivors and 1379 controls confirmed significantly lower CRF among survivors, with a pooled mean difference of −7.1 mL/kg/min^24^. In the St. Jude Lifetime Cohort Study, adult survivors (mean age 35.6 years) demonstrated substantially lower CRF than community controls, particularly among those exposed to cardiotoxic therapies^30^. More than half of survivors met criteria for exercise intolerance ( < 85% of predicted CRF), a condition associated with a fourfold increase in all‑cause mortality over short‑term follow‑up^30^.

Importantly, CRF is not merely a marker of prior treatment exposure but a powerful predictor of future cardiometabolic disease. In a cohort of 2433 survivors from the St. Jude Lifetime Cohort Study, each 1‑metabolic equivalent of task (MET) increase in objectively measured CRF was associated with a 20% reduction in incident cardiometabolic disease^31^. Notably, the greatest benefit was observed among survivors with the lowest baseline fitness levels, identifying CRF as both a mechanistic indicator of cardiometabolic vulnerability and a modifiable target for preventive interventions^31^.

### Metabolic dysregulation and clustered cardiometabolic risk

As survivors enter adulthood, traditional cardiometabolic risk factors—including hypertension, obesity, dyslipidaemia, and impaired glucose metabolism—make an increasingly important contribution to morbidity and mortality^21,32^. In the St. Jude Lifetime Cohort Study, adult survivors exhibited a 2.6‑fold higher prevalence of hypertension than age‑, sex‑, race‑, and BMI‑matched individuals from the general population, with cumulative prevalence exceeding 70% by age 50^33^. Although not all cohorts report similarly elevated rates^33,34^, the overall burden of metabolic abnormalities remains substantial.

Survivors frequently display adverse alterations in body composition, characterised by increased total and central adiposity and reduced lean mass, even when BMI is within the normal range^35,36^. This phenotype is particularly evident among individuals exposed to cranial irradiation or total body irradiation and contributes to insulin resistance, dyslipidaemia, and reduced physical capacity^15,18^.

Two groups—survivors treated with cranial/craniospinal irradiation and those treated with total body irradiation—are at especially high risk of developing the metabolic syndrome^15,16^. Several survivor cohorts exhibit evidence of persistent low-grade systemic inflammation, including higher circulating levels of high-sensitivity C-reactive protein but also of other inflammatory mediators such as IL-6 and TNF-α, which have been associated with adverse cardiometabolic traits^37–39^. Selected cohorts have also reported markers of cellular ageing, including telomere shortening^39^. However, caution is warranted in extrapolating accelerated biological ageing from isolated biomarkers alone. Instead, available evidence supports the presence of a clustered cardiometabolic phenotype, in which metabolic, endocrine, vascular, and inflammatory alterations interact to drive long‑term disease risk.

Recent discoveries have highlighted the role of the gut microbiome—the collective microorganisms (bacteria, archaea, fungi, and viruses) residing in the gastrointestinal tract—in health and disease, including cardiometabolic conditions. In long-term survivors of childhood malignancies (aged 10–40 years, ≥5 years post-treatment), a lower microbiome alpha-diversity was observed compared with controls, alongside differences in the abundance of specific bacterial taxa^40^. Survivors who had received radiation to the central nervous system or abdomen/pelvis had even lower alpha-diversity than those who had not^40^. Another exploratory study found no differences in the alpha- or beta-diversity of the microbiome between survivors of acute lymphoblastic leukaemia (aged <19 years, ≥1 year post-treatment) and their healthy siblings^41^. Yet, the genus *Faecalibacterium—*a major butyrate-producing taxon known to inhibit activation of the nuclear factor kappa B inflammatory pathway and to promote the production of anti-inflammatory cytokines—was depleted in survivors, mirroring microbial shifts observed in obesity^41^. It was hypothesised that such alterations in leukaemia survivors begin in childhood and may contribute to increased susceptibility to chronic illness later in life^41^. Physical activity for 8 weeks combined with probiotics improved the gut microbiota composition of leukaemia survivors (aged 8.9 ± 3.3 years, 1–3 years after completion of treatment), increasing microbiome alpha-diversity and the relative abundance of *Lactobacillus casei* and the genus *Veillonella*^42^, which reduced the susceptibility to type 2 diabetes^43^ and enhanced exercise performance^44^, respectively.

## Modifiers of cardiometabolic risk beyond treatment exposure

### Ethnicity‑related disparities

**Fig. 3:**
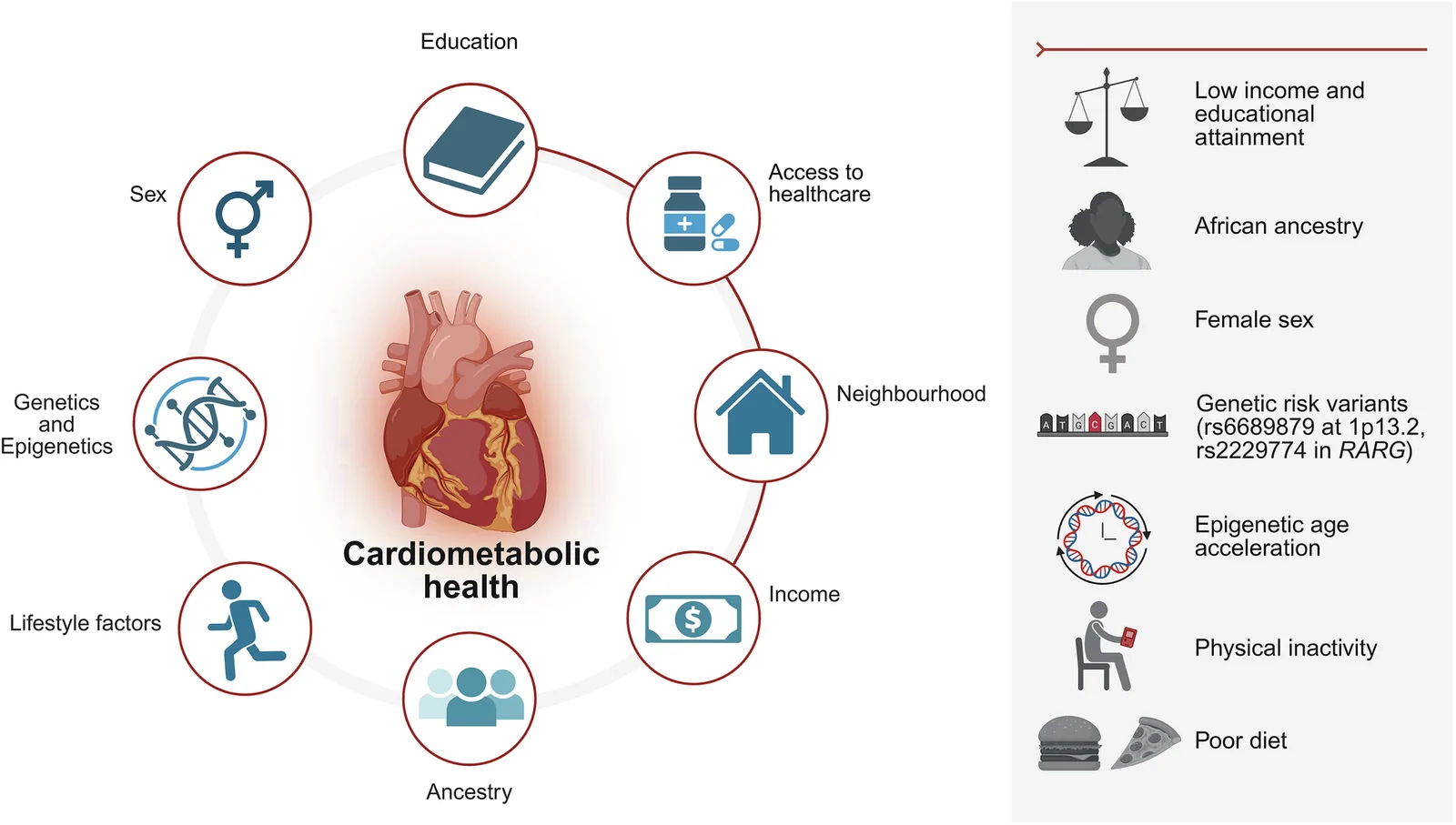
Key determinants of cardiometabolic health, beyond cancer treatment-related exposures, in survivors of cancer diagnosed during childhood or adolescence. These determinants include genetic, epigenetic, biological, social, and lifestyle-related factors that jointly shape long-term cardiometabolic risk. Genetic and epigenetic susceptibility includes risk variants (e.g., rs6689879 at 1p13.2 and rs2229774 in *RARG*) and epigenetic age acceleration, which may modulate susceptibility to treatment‑related cardiometabolic injury. The left-hand panel shows the domains that shape cardiometabolic health; the right-hand panel lists the specific factors within these domains that have been associated with higher cardiometabolic risk in this population. This figure was created with BioRender. RARG retinoic acid receptor gamma.

Ethnic background influences cardiometabolic outcomes among survivors of childhood cancer (Fig. 3), although the magnitude and direction of these associations vary across cohorts and age at diagnosis. In a cohort of survivors diagnosed between ages 0–14 years, cardiometabolic disease-related mortality did not differ significantly between African American and European American individuals (hazard ratio [HR], 1.08; 95% confidence interval [CI], 0.62–1.89)^45^. However, among survivors diagnosed during adolescence or young adulthood (ages 15–39 years), African American individuals experienced significantly higher cardiometabolic risk compared with their European American counterparts, even after adjustment for demographics, socioeconomic factors, and cancer type (HR, 1.55; 95% CI, 1.33–1.81)^46^.

Within the Childhood Cancer Survivor Study, non‑Hispanic African American survivors exhibited a higher incidence of severe congestive heart failure (grade 3–5), an association that was attenuated but not abolished after adjusting for socioeconomic status and cardiometabolic risk factors^47^. Similarly, in the St. Jude Lifetime Cohort Study, survivors of African ancestry exposed to cardiotoxic treatments had markedly higher odds of developing moderate‑to‑severe cardiomyopathy than those of European ancestry, independent of treatment intensity and clinical risk factors^48^. Collectively, these findings suggest that ethnic disparities in cardiometabolic outcomes reflect a complex combination of genetic susceptibility, treatment exposure, and structural determinants of health.

### Genetic and epigenetic susceptibility

In the St. Jude Lifetime Cohort Study, the rs6689879 variant on chromosome 1p13.2 was associated with a fivefold increased risk of cardiomyopathy among survivors of African ancestry, compared with a 1.3-fold increase in those of European ancestry^48^. This association was attributed to hypomethylation in the promoter region of the putative homeodomain transcription factor 1 *(PHTF1*) gene, leading to upregulation of gene expression in response to doxorubicin-induced cardiotoxicity^48^. Another candidate marker of increased susceptibility to anthracycline-induced cardiotoxicity is the nonsynonymous variant rs2229774 (p.Ser427Leu) in the retinoic acid receptor gamma (*RARG*) gene^7^, potentially via downregulation of cardioprotective enzymes (e.g., topoisomerase IIβ) and disruption of the cardioprotective extracellular signal-regulated kinase pathway^8^. Amongst survivors of childhood cancer of European ancestry, the CUGBP Elav-Like Family Member 4 (*CELF4*) rs1786814 GG genotype was associated with a tenfold higher risk of anthracycline-related cardiomyopathy compared with AA/GA genotypes, possibly due to impaired regulation of Nav1.6, a sodium channel involved in cardiac electrophysiology^49^. Other variants associated with increased susceptibility to anthracycline-induced cardiotoxicity in children include rs17863783 in the UDP-glucuronosyltransferase (*UGT1A6*) gene and rs7853758 in the solute carrier family 28-member 3 (*SLC28A3*) gene^50^.

Beyond inherited genetic variation, epigenetic mechanisms appear to play a central role in shaping long‑term cardiometabolic risk. In the St. Jude Lifetime Cohort Study, epigenetic age acceleration—defined as discordance between DNA methylation-derived biological age and chronological age—partially mediated the associations between cancer treatments and multiple cardiometabolic outcomes, including hypertension, abnormal glucose metabolism, obesity, cardiomyopathy, and myocardial infarction^51^. Epigenome‑wide analyses further identified DNA methylation sites linked to cardiometabolic risk factors in nearly 3000 survivors, implicating pathways related to inflammation, lipid metabolism, and mitochondrial function^52^. These findings support a model in which cancer therapy leaves a durable epigenetic imprint that contributes to accelerated cardiometabolic ageing.

### Sex‑specific vulnerability

Evidence suggests sex‑related differences in susceptibility to treatment‑related cardiometabolic injury, although findings are not fully consistent across studies. Early clinical investigations showed that females treated with anthracyclines during childhood had a higher risk of early‑onset cardiotoxicity than males^53^. Subsequent studies reported greater long‑term cardiovascular risk in female survivors, including higher rates of heart failure and cardiac mortality following anthracycline exposure^54,55^.

Biological mechanisms underlying this vulnerability may include sex‑dependent differences in anthracycline pharmacokinetics, myocardial oxidative stress responses, and hormonal modulation of cardioprotective pathways. Experimental and clinical evidence suggests that oestrogens may influence anthracycline‑induced cardiotoxicity, potentially offering protection in some contexts while exacerbating injury under others^56,57^. However, not all large cohort studies report consistent sex differences^57^, indicating that sex interacts with age at exposure, treatment intensity, and cardiometabolic co‑factors.

Sex‑specific differences have also been observed in functional outcomes. Among long‑term survivors of Hodgkin lymphoma treated with chest radiotherapy, women reached clinically abnormal values of cardiorespiratory fitness decades earlier than men, a finding of particular importance given the strong association between reduced fitness and subsequent cardiovascular events^58^. These data indicate the need for sex‑informed risk stratification in survivorship care.

### Social and structural determinants of cardiometabolic health

Social and structural determinants exert a profound influence on cardiometabolic outcomes among childhood cancer survivors. US survivors residing in higher‑income neighbourhoods or areas with greater physician density were more likely to receive cardiology referral and echocardiographic screening^59^. In contrast, survivors from socioeconomically disadvantaged backgrounds were more likely to lack health insurance and to present with multiple cardiometabolic risk factors, including obesity and diabetes^60^.

Residence in socioeconomically deprived neighbourhoods has been associated with higher rates of cardiovascular events and mortality among adolescent and young adult cancer survivors, independent of ethnicity^46^. Similar gradients have been observed in population‑based cohorts, where increasing levels of poverty were linked to higher cardiometabolic disease mortality^61^. Within the St. Jude Lifetime Cohort Study, survivors living in disadvantaged census‑block groups experienced higher late mortality from both health‑related and all‑cause outcomes^32^. These findings highlight that equitable survivorship care must address not only biological risk but also barriers to preventive services, health literacy, and long‑term follow‑up.

### Lifestyle determinants of cardiometabolic health

**Fig. 4:**
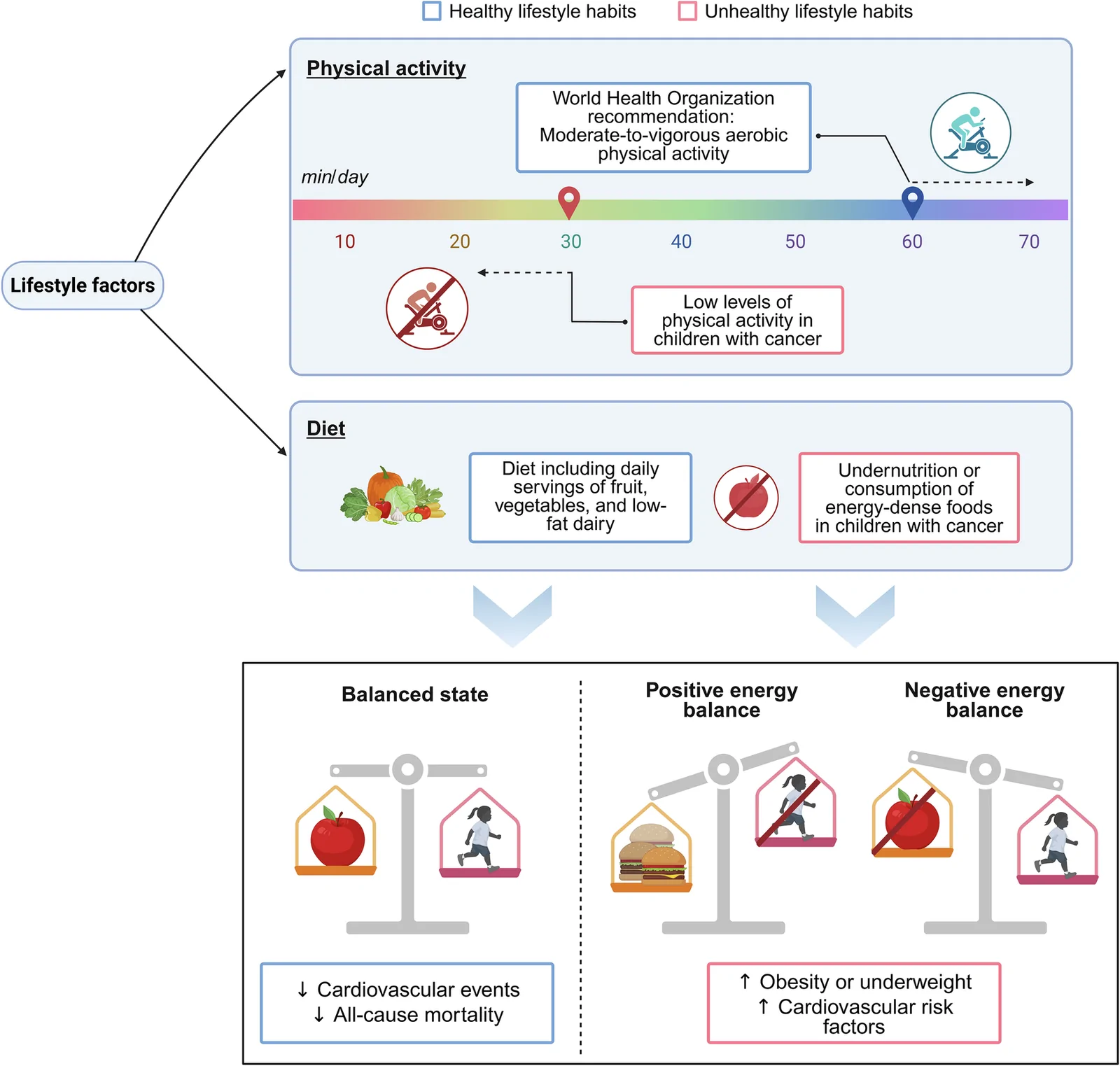
Lifestyle habits (physical activity and nutrition) in patients with (or survivors of) childhood/adolescent cancer, contrasting healthy and unhealthy patterns and their associated outcomes. A healthy lifestyle and balanced energy state are associated with fewer cardiovascular events and lower all‑cause mortality, whereas physical inactivity and energy imbalance promote adiposity (or, in some patients, undernutrition) and associated cardiometabolic risk factors. This figure was created with BioRender.

The WHO recommends that children/adolescents aged 5–17 years—including those with disabilities—perform moderate-to-vigorous physical activity (MVPA), primarily of aerobic nature, 60 or more minutes per day to support optimal cardiometabolic health and overall development (Fig. 4)^62^. However, these targets are rarely achieved in the context of childhood cancer, beginning as early as the treatment phase. For instance, data from Spanish children (mean age, 10–11 years) undergoing intensive therapy for solid tumours indicated median MVPA levels ≤ 30 min per day^63^. Similarly, North American adolescents in active follow-up after treatment for various cancer types reported daily MVPA durations of ~26 min^64^. A recent study reported that the magnitude of the cardiometabolic disease incidence attributable to insufficient physical activity levels in US survivors of Hodgkin lymphoma was approximately 12-fold higher than that observed in sibling controls^65^. Although more research is needed—especially using larger samples and high-quality randomised controlled trial designs—and despite heterogeneity between studies, supervised exercise interventions (typically combining aerobic and resistance training) during and/or after treatment in children or adolescents with cancer may have cardioprotective effects, including preservation of left-ventricular ejection fraction^66^ and improvements in CRF^67^.

Physical inactivity remains a major concern among survivors, as it independently contributes to morbidity and mortality. In a study with a median follow-up of 11.9 years involving 1187 5-year survivors of Hodgkin lymphoma (median age, 31.2 years), engaging in vigorous physical activity (≥9 MET-hour/week) was associated with a 51% reduction in the risk of major cardiovascular events^68^. Similarly, in a large cohort (*n* = 15,450) of US survivors diagnosed between 1970–1986, a dose-response reduction in mortality was observed over 15 years, including a 40% reduction in all-cause mortality over 8 years among survivors who engaged in ≥ 7.9 MET-hour/week^69^. Adoption of a healthy lifestyle—defined as regular activity, as well as abstinence from smoking and heavy alcohol use, and maintenance of a normal BMI—was associated with a 20% reduction in late mortality risk in a large, multi-institutional cohort of North American survivors, independent of prior cancer treatment exposures^21^.

Optimal nutritional status is essential for healthy growth and development in children and remains central to adult health by influencing long-term cardiometabolic risk^70^. Children with cancer often face a dual burden of malnutrition^71^: high-intensity treatments can lead to undernutrition requiring complex nutrition support^72^, while conditions like standard-risk acute lymphoblastic leukaemia may trigger increased appetite and cravings for energy-dense food^73^. Despite the importance of this developmental period, over 90% of patients consume poor-quality diets low in vegetables^74^, and 38% have obesity by the end of treatment^71^. These patterns persist in survivorship, with observational data showing high prevalence of Western-style dietary patterns linked to increased cardiometabolic risk^75^. Higher fruit and vegetable intake was associated with maintaining a healthy BMI over the 1-year follow-up among childhood cancer survivors (approximately 47% of participants)^76^.

To date, there is a paucity of evidence on diet interventions targeting cardiometabolic disease risk factors for childhood cancer survivors. In a 24-month phase III trial, 358 survivors of acute lymphoblastic leukaemia with overweight (median age 37 years) were randomised to a remote lifestyle intervention (phone coaching plus website) or a control group receiving mailed brochures^77^. The intervention targeted ≥5% weight loss (mean difference: 2.75 kg) via a low-energy, low-sodium diet (7–12 daily servings of fruit, vegetables, and low-fat dairy) and ≥180 min per week of MVPA^77^. At 24 months, there were no differences between groups in weight loss or cardiometabolic risk profile (blood pressure, HDL/LDL-cholesterol, or triglycerides)^77^. Adherence was low: 25% of participants never completed a coaching call, and few completed ≥80% of sessions^77^. Given the long treatment duration and the young age at diagnosis—when dietary habits are still forming—earlier interventions during cancer treatment are needed to prevent cardiometabolic disease risk factors such as obesity in later life.

## Conclusions and future directions

Survivors of childhood cancer face a uniquely elevated risk of cardiometabolic disease driven primarily by treatment‑related injury occurring during critical windows of cardiovascular and metabolic development. Although survival rates have improved dramatically, cardiometabolic complications remain a major contributor to long‑term morbidity and mortality (see also Table 1 for a summary).

**Table 1:** Summary of major cardiometabolic phenotypes in survivors of childhood cancer

Cardiometabolic domain	Key clinical findings in survivors	Major contributing mechanisms	Representative evidence	Clinical implications
Accelerated cardiometabolic ageing and mortality	Earlier onset of CVD and excess late mortality compared with the general population	Early treatment-related biological injury interacting with ageing processes and acquired CVD risk factors	CCSS and St. Jude Lifetime Cohort studies^21–23^	Need for lifelong surveillance and early prevention strategies
Cardiac dysfunction	Subclinical systolic dysfunction, autonomic abnormalities, increased risk of cardiomyopathy and heart failure	Anthracycline-induced myocardial injury; radiotherapy-related fibrosis and microvascular damage	Echocardiographic and cohort evidence^24–26^	Early detection using imaging and circulating biomarkers
Cardiorespiratory fitness	Low VO_2peak_ and high prevalence of exercise intolerance	Cardiac injury, skeletal muscle impairment, physical inactivity, and autonomic dysfunction	Meta-analyses and St. Jude Lifetime Cohort^24,29–31^	Key integrative risk marker and modifiable target
Hypertension	Markedly increased prevalence with ageing	Vascular injury, renal effects of therapy, endocrine alterations	St. Jude Lifetime Cohort and DCCSS-LATER^33,34^	Early screening and aggressive risk-factor control
Body composition abnormalities	Increased adiposity and reduced lean mass despite normal BMI	Cranial/total body irradiation, endocrine disruption, inactivity	Long-term cohort studies^35,36^	Assessment beyond BMI; muscle preservation strategies
Metabolic syndrome	High prevalence, particularly after cranial or total body irradiation	Hypothalamic–pituitary axis dysfunction, insulin resistance	Guidelines and Dutch survivor cohorts^15,16^	Risk-based metabolic surveillance
Low-grade inflammation	Elevated inflammatory biomarkers in selected cohorts	Interaction of adiposity, metabolic dysregulation, and vascular injury	Observational survivor studies^39^	Contributor to clustered cardiometabolic risk

*BMI* body mass index, *CCSS* Childhood Cancer Survivor Study, *CVD* cardiovascular disease, *DCCSS-LATER* Dutch Childhood Cancer Survivor Study-Late Effects, *VO*_*2peak*_ peak oxygen uptake.

Childhood cancer survivorship differs fundamentally from adult‑onset cancer. In adults, cardiometabolic risk factors are often present at diagnosis and interact with treatment toxicity in an established preventive care context. In contrast, childhood cancer survivors are typically exposed to cardiotoxic therapies early in life, with cardiometabolic disease emerging years or decades later in otherwise young, asymptomatic individuals. This prolonged temporal dissociation between exposure and clinical disease places prevention, early risk identification, and longitudinal surveillance at the centre of survivorship care. Independent of treatment, the malignancy itself may condition cardiometabolic health, as tumour-related systemic inflammation, cachexia, and immune dysregulation have been associated with adverse cardiometabolic and cardiovascular profiles^78,79^. On the other hand, longitudinal surveillance might be particularly important to prevent the occurrence of cancer during adulthood in those childhood cancer survivors with cardiovascular disease. Indeed, there is mounting clinical and preclinical evidence that cardiovascular disease can itself accelerate tumour growth and metastasis (the so-called *reverse cardio-oncology*) through several mechanisms, including shared risk factors, reprogramming of immune responses, changes in gene expression, and the release of cardiac factors that result in selective advantages for tumour cells or their local milieu^78^. Furthermore, given that emerging data suggest concurrent molecular alterations in both cancer and cardiovascular diseases (which might partly explain the co-occurrence of these diseases), therapies (e.g., angiogenesis inhibitors and anthracyclines) may also act on the same intracellular metabolic targets^79^. Thus, future oncological and cardiological therapies should keep these interdisciplinary effects in mind, to minimise adverse effects and reduce mortality and morbidity^79^.

The knowledge of the specific molecular alterations may lead to personalised treatment options in cancer therapies and to personalised cardiac surveillance protocols, according to cancer type and previous metabolomic profiles. Emerging evidence supports a shift towards multimodal, biomarker‑guided risk stratification, integrating circulating biomarkers (e.g., NT‑proBNP), imaging‑derived parameters (e.g., GLS), and genomic or epigenetic risk indicators to identify high‑risk survivors before overt disease develops. Stratified surveillance approaches may enable earlier intervention and more efficient allocation of resources.

Lifestyle factors represent an important modifiable target across survivorship. Improvements in cardiorespiratory fitness, physical activity, and nutritional quality have the potential to attenuate treatment‑related cardiometabolic risk, even decades after exposure. Given that childhood and adolescence are formative periods for lifelong health behaviours, preventive strategies initiated during or shortly after treatment may yield disproportionate long‑term benefits.

Finally, addressing social and structural determinants of health is essential to ensure equitable access to preventive strategies and cardiometabolic screening. A multidisciplinary survivorship model integrating cardio‑oncology, endocrinology, rehabilitation, and public health perspectives is required to improve long‑term cardiometabolic outcomes among individuals diagnosed with cancer during childhood or adolescence.

## Data Availability

No datasets were generated or analysed during the current study.
